# A Mathematical Model for the Reciprocal Differentiation of T Helper 17 Cells and Induced Regulatory T Cells

**DOI:** 10.1371/journal.pcbi.1002122

**Published:** 2011-07-28

**Authors:** Tian Hong, Jianhua Xing, Liwu Li, John J. Tyson

**Affiliations:** 1 Genetics, Bioinformatics, and Computational Biology Program, Virginia Polytechnic Institute and State University, Blacksburg, Virginia, United States of America; 2 Department of Biological Sciences, Virginia Polytechnic Institute and State University, Blacksburg, Virginia, United States of America; Emory University, United States of America

## Abstract

The reciprocal differentiation of T helper 17 (T_H_17) cells and induced regulatory T (iT_reg_) cells plays a critical role in both the pathogenesis and resolution of diverse human inflammatory diseases. Although initial studies suggested a stable commitment to either the T_H_17 or the iT_reg_ lineage, recent results reveal remarkable plasticity and heterogeneity, reflected in the capacity of differentiated effectors cells to be reprogrammed among T_H_17 and iT_reg_ lineages and the intriguing phenomenon that a group of naïve precursor CD4^+^ T cells can be programmed into phenotypically diverse populations by the same differentiation signal, transforming growth factor beta. To reconcile these observations, we have built a mathematical model of T_H_17/iT_reg_ differentiation that exhibits four different stable steady states, governed by pitchfork bifurcations with certain degrees of broken symmetry. According to the model, a group of precursor cells with some small cell-to-cell variability can differentiate into phenotypically distinct subsets of cells, which exhibit distinct levels of the master transcription-factor regulators for the two T cell lineages. A dynamical control system with these properties is flexible enough to be steered down alternative pathways by polarizing signals, such as interleukin-6 and retinoic acid and it may be used by the immune system to generate functionally distinct effector cells in desired fractions in response to a range of differentiation signals. Additionally, the model suggests a quantitative explanation for the phenotype with high expression levels of both master regulators. This phenotype corresponds to a re-stabilized co-expressing state, appearing at a late stage of differentiation, rather than a bipotent precursor state observed under some other circumstances. Our simulations reconcile most published experimental observations and predict novel differentiation states as well as transitions among different phenotypes that have not yet been observed experimentally.

## Author Summary

In order to perform complex functions upon pathogenic challenges, the immune system needs to efficiently deploy a repertoire of specialized cells by inducing the differentiation of precursor cells into effector cells. In a critical process of the adaptive immune system, one common type of precursor cell can give rise to both T helper 17 cells and regulatory T cells, which have distinct phenotypes and functions. Recent discoveries have revealed a certain heterogeneity in this reciprocal differentiation system. In particular, treating precursor cells with a single differentiation signal can result in a remarkably diverse population. An understanding of such variable responses is limited by a lack of quantitative models. Our mathematical model of this cell differentiation system reveals how the control system generates phenotypic diversity and how its final state can be regulated by various signals. The model suggests a new quantitative explanation for the scenario in which the master regulators of two different T cell lineages can be highly expressed in a single cell. The model provides a new framework for understanding the dynamic properties of this type of regulatory network and the mechanisms that help to maintain a balance of effector cells during the inflammatory response to infection.

## Introduction

CD4^+^ T cells are important components of the adaptive immune system in higher vertebrates. By producing various cytokines, they perform critical functions such as helping B cells to produce antibodies, activating CD8^+^ cytotoxic T cells, enhancing the innate immune system, and suppressing the immune response to avoid autoimmunity [1], [2], [3]. In peripheral tissues, such as lymph nodes, blood and sites of infection, antigen-inexperienced (naïve) CD4^+^ T cells can differentiate into effector cells of specialized phenotypes upon stimulation by cognate antigen delivered to the T cell receptor by Antigen Presenting Cells (APCs). Proliferation and differentiation of activated naïve T cells depends on their particular cytokine microenvironment. These specialized effector T cells produce distinct cytokine profiles tailored for their specialized functions. Also, they express lineage-defining transcription factors (“master regulators”). In general, high expression level of a particular master regulator is observed only in cells of a particular lineage, and the overexpression of a particular master regulator induces the production of the corresponding lineage-defining cytokines [4], [5].

The fate of a naïve CD4^+^ T cell was traditionally thought to be either T helper 1 (T_H_1) cell or T helper 2 (T_H_2) cell [6]. In the last decade, a third type of T helper cell (T_H_17), derived from naïve CD4^+^ T cells, was discovered [7]. T_H_17 cells produce interleukin-17A (IL-17A), IL-17F and IL-22 as their lineage-defining cytokines, and the retinoic acid receptor-related orphan receptor gamma t (RORγt) transcription factor is considered the master regulator of this lineage [8], [9]. In addition, naïve CD4^+^ T cells were found to be able to differentiate into a fourth lineage of (regulatory) T cells, which were called induced regulatory T (iT_reg_) cells to distinguish them from natural regulatory T (nT_reg_) cells, which differentiate in the thymus instead of the periphery [10]. iTreg cells are characterized by producing IL-10 and transforming growth factor-β (TGF-β) and highly expressing forkhead box P3 (Foxp3) transcription factor as their master regulator [11]. T_H_17 cells are pro-inflammatory because they secret cytokines that promote inflammation, whereas iT_reg_ cells are anti-inflammatory because their lineage-defining cytokines can reduce the inflammatory response.

The differentiation pathways of naïve T cells into T_H_17 and iT_reg_ lineages are closely related. First, stimulation by TGF-β is necessary for the differentiation of both lineages [12]. The differentiation of T_H_17 and iT_reg_ cells are reciprocally regulated in the presence of TGF-β, i.e. inhibiting the differentiation pathway of one lineage will result in activation of the pathway for the other lineage. This is due to the mutual antagonism between RORγt and Foxp3. Furthermore, polarizing signals, such as IL-6 and retinoic acid, can induce the differentiation of one lineage and repress that of the other one [12]. Nonetheless, differentiated iT_reg_ cells can be reprogrammed into T_H_17 cells in an appropriate cytokine environment [13], suggesting significant plasticity of these two lineages. In addition, stable co-expression of their master regulators (RORγt and Foxp3) is observed both *in vivo* and *in vitro* [14], [15]. Interestingly, these double-expressing cells were found to possess either regulatory or dual (regulatory and proinflammatory) functions *in vivo* [14], [15].

**Figure 1:**
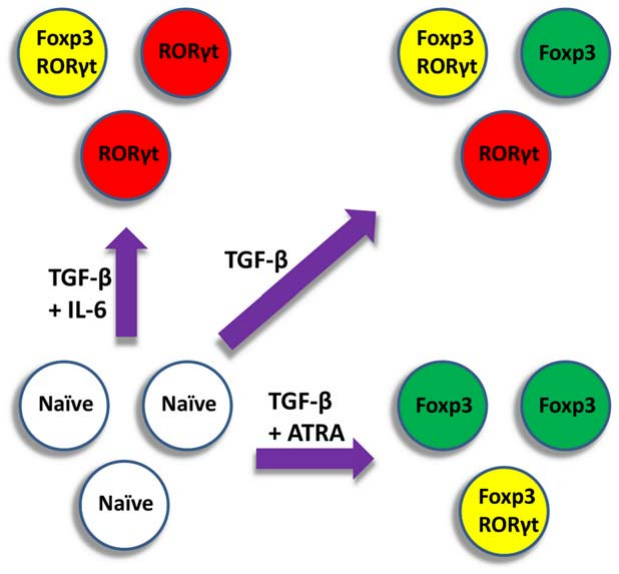
Induction of differentiation from naïve CD4^+^ T cells to T_H_17 and iT_reg_. A population of antigen-activated naïve CD4^+^ T cells (white) can be induced by different types of cytokine micro-environment to produce corresponding differentiated cell populations. T_H_17 cells (red) express the RORγt transcription factor, and iT_reg_ cells (green) express the Foxp3 transcription factor. Some cells (yellow) express both master regulators and may possess both regulatory and pro-inflammatory functions.

Perhaps the most intriguing phenomenon is that antigen-activated naïve CD4^+^ T cells treated with TGF-β alone give rise to a heterogeneous population, which may include three phenotypes (Foxp3-only, RORγt-only, and double-expressing cells) at an intermediate TGF-β concentration [16], or two phenotypes (RORγt-only and double-expressing cells) at a higher TGF-β concentration [15]. In combination with TGF-β, IL-6 can induce the differentiation of RORγt expressing cells, whereas all*-trans* retinoic acid (ATRA) can induce the differentiation of Foxp3 expressing cells [16], [17] (Figure 1). All of these *in vitro* derived phenotypes can be observed *in vivo*, and at least some of their respective functions have been demonstrated, suggesting that these *in vitro* differentiation assays provide important clues to our understanding of the development of T_H_17 and iT_reg_ cells in the body.

Mathematical modeling has contributed to our understanding of the differentiation of T_H_1 and T_H_2 cells [18], [19], [20], [21], [22], [23], [24]. Höfer et al. first demonstrated that the dynamics of the key transcription factors can govern the robustness of the lineage choice and maintenance [18], [19]. Yates et al. later related transcription factor dynamics to the mix of T_H_1 and T_H_2 cells in a population of differentiating T cells [20]. Recently, Bonneau et al. [25] have proposed a Boolean-network model of the comprehensive repertoire of CD4^+^ T cell phenotypes, including T_H_17 and iT_reg_ cells. Drawing inspiration from these earlier models, we have sought to explain, with a computational model, the remarkable heterogeneity of the T_H_17-iT_reg_ reciprocal-differentiation system.

In terms of this model, we show that a population of naïve CD4^+^ T cells, with some small cell-to-cell variability, can differentiate into a heterogeneous population of effector cells with distinct phenotypes upon treatment with the primary differentiation signal (TGF-β). Polarizing signals, such as IL-6 and ATRA, can skew the differentiation to one or two phenotypes. A control system with these properties can generate functional diversity of the induced cell populations and can be regulated with great flexibility by diverse environmental cues. In addition, the model suggests how treatment with different concentrations of TGF-β may favor different responding phenotypes, and how conversions among these phenotypes may be guided. Finally, the model gives a new quantitative explanation for double-expressing cells, suggesting that they are ‘re-stabilized co-expressing’ cells rather than transient intermediate cells in the differentiation pathway. The model predicts that double-expressing cells should appear at a relatively late stage of the differentiation process, and they may be intended for specific functions. In all, our model provides a novel mathematical framework for understanding this reciprocal differentiation system, and it gives new insights into the regulatory mechanisms that underlie the molecular control of certain immune responses.

## Results

### A model with symmetrical interactions predicts three differentiated phenotypes of CD4^+^ T cells induced by TGF-β

**Figure 2:**
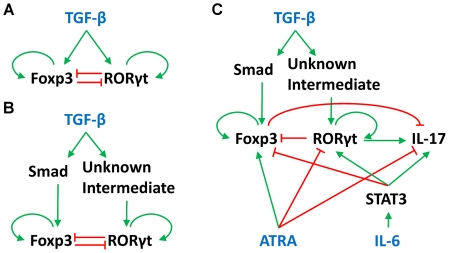
Influence diagrams of the mathematical models. **A.** Symmetrical model without intermediates. **B.** Symmetrical model with intermediates. **C.** Asymmetrical model with three input signals: TGF-β, ATRA, and IL-6.

**Figure 3:**
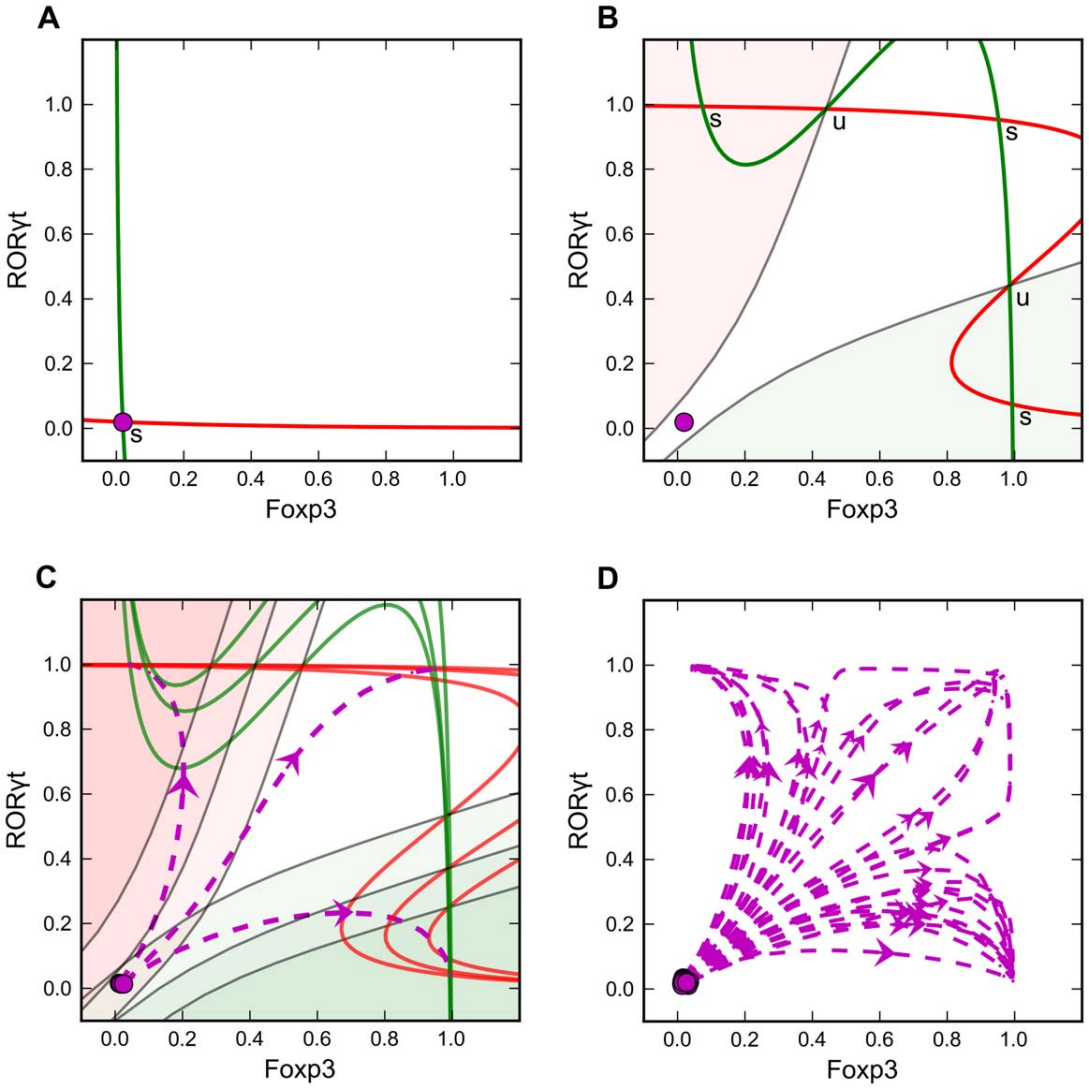
Phase plane analysis of the symmetrical model without intermediates. X and Y axes: dimensionless quantities that represent the intracellular concentrations of master regulators Foxp3 and RORγt respectively. Value = 1 indicates the maximum intracellular concentration of the master regulator, and value = 0 indicates the absence of the master regulator. Red Line: nullcline for RORγt. Green line: nullcline for Foxp3. Steady states, at the intersections of red and green nullclines, are labeled as ‘u’ (unstable) or ‘s’ (stable). Magenta dashed line with arrow: trajectory of a time-course simulation. Semi-transparent red and green areas: the basins of attractions for RORγt^high^Foxp3^low^ and RORγt^low^Foxp3^high^ states, respectively. **A.** Phase plane for the average cell with [TGF-β] = 0. Magenta circle: RORγ^low^Foxp3^low^ steady state. **B.** Phase plane for the average cell with [TGF-β] = 0.5 units. Magenta circle is the location of the steady state in Panel A. **C.** Overlaid phase planes and trajectories for three cells adopting distinct fates. **D.** Simulation trajectories for a population of 30 cells on the plane of RORγt and Foxp3.

To illustrate our basic idea, we first construct a model of a simple and perfectly symmetrical regulatory network (Figure 2A). In the Methods section we describe how this network is converted into a pair of nonlinear ordinary differential equations (ODEs) for the time rates of change of Foxp3 and RORγt. The rate functions for this model contain 12 kinetic parameters, whose basal values are specified in the Methods section (Table 1) for the “symmetrical model without intermediates”. The solution of these ODEs for the basal values, and with [TGF-β] = 0, evolves to a stable steady state where both RORγt and Foxp3 have a low level of expression (RORγt^low^Foxp3^low^). This steady state corresponds to a naïve CD4^+^ T cell (Figure 3A). In the presence of a sufficient TGF-β signal, the regulatory network might evolve to one of three other steady states, namely RORγt^high^Foxp3^low^, RORγt^low^Foxp3^high^ and RORγt^high^Foxp3^high^ states, corresponding to RORγt-only, Foxp3-only and double-expressing phenotypes. Note that these stable steady states are also referred as ‘cell fate attractors’ in some other studies, and this concept facilitates our understanding of cell lineage choice and reprogramming (reviewed in [26]). Figure 3B shows a scenario in which the TGF-β signal triggers the formation of a tri-stable system. In this particular case, the RORγt^low^Foxp3^low^ state is no longer a stable steady state, and naïve cell, which was previously stabilized in the RORγt^low^Foxp3^low^ state, would differentiate into the RORγt^high^Foxp3^high^ state, whose basin of attraction (the white region in Figure 2B) contains the naïve state of the cell.

**Table 1:** Descriptions and basal values of parameters.

Parameter name	Description	Basal value in symmetrical model without intermediates	Basal value in symmetrical model with intermediates	Basal value in model with broken symmetry
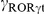	Relaxation rate of RORγt	1	1	1
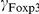	Relaxation rate of Foxp3	1	1	1
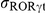	Steepness of sigmoidal function for RORγt	5	5	7
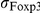	Steepness of sigmoidal function for Foxp3	5	5	5
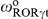	Basal activation state of RORγt	−0.8	−0.8	−0.84
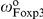	Basal activation state of Foxp3	−0.8	−0.8	−0.92
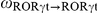	Weight of autoactivation of RORγt	1.24	1.2	0.7
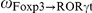	Weight of inhibition on RORγt by Foxp3	−0.4	−0.4	NA
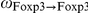	Weight of autoactivation of Foxp3	1.24	1.2	1.28
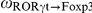	Weight of inhibition on Foxp3 by RORγt	−0.4	−0.4	−0.54
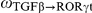	Weight of activation on RORγt by TGF-β	1.2	NA	NA
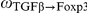	Weight of activation on Foxp3 by TGF-β	1.2	NA	NA
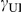	Relaxation rate of unknown intermediate (UI)	NA	1	1
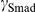	Relaxation rate of Smad	NA	1	1
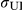	Steepness of sigmoidal function for UI	NA	10	12
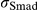	Steepness of sigmoidal function for Smad	NA	10	20
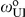	Basal activation state of UI	NA	−0.2	−0.23
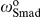	Basal activation state of Smad	NA	−0.2	−0.225
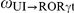	Weight of activation on RORγt by UI	NA	0.62	0.86
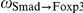	Weight of activation on Foxp3 by Smad	NA	0.62	0.68
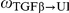	Weight of activation on UI by TGF-β	NA	1.2	1
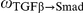	Weight of activation on Smad by TGF-β	NA	1.2	1
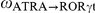	Weight of inhibition on RORγt by ATRA	NA	NA	−0.04
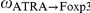	Weight of activation on Foxp3 by ATRA	NA	NA	0.035
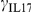	Relaxation rate of IL-17	NA	NA	1
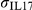	Steepness of sigmoidal function for IL-17	NA	NA	30
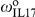	Basal activation state of IL-17	NA	NA	−0.82
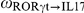	Weight of activation on IL-17 by RORγt	NA	NA	0.22
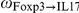	Weight of inhibition on IL-17 by Foxp3	NA	NA	−0.8
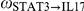	Weight of activation on IL-17 by STAT3	NA	NA	0.6
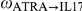	Weight of inhibition on IL-17 by ATRA	NA	NA	−0.1
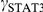	Relaxation rate of STAT3	NA	NA	0.1
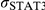	Steepness of sigmoidal function for STAT3	NA	NA	10
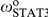	Basal activation state of STAT3	NA	NA	−0.4
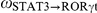	Weight of activation on RORγt by STAT3	NA	NA	0.2
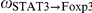	Weight of inhibition on Foxp3 by STAT3	NA	NA	−0.1
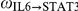	Weight of activation on STAT3 by IL-6	NA	NA	0.2
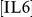	Concentration of IL-6	NA	NA	C
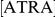	Concentration of ATRA	NA	NA	C
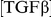	Concentration of TGF-β	C	C	C

C: Values are specified in each simulation and might be changed at certain times during the simulation. These parameters are not subject to cell-to-cell variations.

However, cell-to-cell variability can produce other results. We interpret cell-to-cell-variability as small deviations of the parameter values from their basal settings in Table 1. The basal settings correspond to the behavior of an “average” cell, but any particular cell will deviate somewhat from this average behavior. As consequences of the changing parameter values in any particular cell, the position of the RORγt^low^Foxp3^low^ state changes, the boundaries of the basins of attractions change, and the fate of the naïve cell may change. The naïve T cell will differentiate into the stable steady state in whose basin of attraction it lies. That is, depending on the precise parameter values of the cell, its RORγt^low^Foxp3^low^ state may lie in any of the three basins of attraction of the TGF-β-stimulated system. Figure 3C depicts three cells in the population that adopt three different fates because of the variability among them. With a random sample of cells, each of the three differentiated states can be populated by a significant fraction of cells (Figure 3D). Although cell-to-cell variability does not make large changes in the position of the RORγt^low^Foxp3^low^ state, it has a dramatic influence on the basins of attraction of the stable steady states, which determines the fate of the cell once the differentiation signal is turned on.

**Figure 4:**
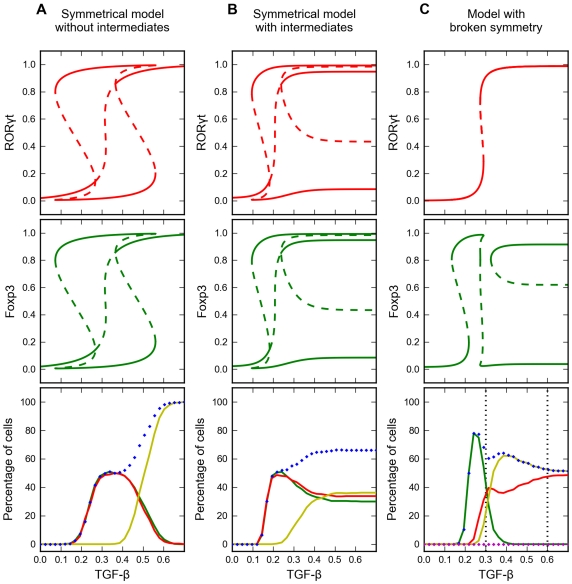
Bifurcation diagrams and signal-response curves for three models. Upper and middle panels: one-parameter bifurcation diagrams for the average cell. Steady state levels of RORγt and Foxp3 are plotted as functions of TGF-β concentration. Solid line: stable steady states. Dashed line: unstable steady states. Lower panels: signal-response curves. For each point on the abscissa (for [TGF-β] = constant), we simulate induced differentiation of a population of 1000 cells. Percentages of cells at the alternative steady states are plotted as functions of TGF-β concentration used for induction. Red line: RORγt-only cells. Green line: Foxp3-only cells. Yellow line: double-expressing cells. Blue marker: Foxp3-expressing cells. Magenta marker: IL-17 producing cells. **A.** Symmetrical model without intermediates. **B.** Symmetrical model with intermediates. **C.** Asymmetric model. Dotted vertical lines denote representative experimental levels of TGF-β.

Since the system has four distinct steady states that correspond to four distinct phenotypes, we next looked for the relationships among these steady states using bifurcation analysis of an average cell. Because of the symmetrical nature of the interactions, an average cell exhibits sub-critical pitchfork bifurcations with TGF-β concentration as the control parameter (Figure 4A). (The notion of a pitchfork bifurcation was used earlier, in references [27], [28], to explain a system of hematopoietic cell differentiation in which multiple lineages might be adopted.) Notably, the RORγt^low^Foxp3^low^ state is only stable at low TGF-β concentration. At an intermediate concentration of TGF-β (∼0.25 units in Figure 3A), the system bifurcates into two lineage-specific branches, corresponding to RORγt^high^Foxp3^low^ and RORγt^low^Foxp3^high^ states. The fourth type of stable steady state (RORγt^high^Foxp3^high^) appears at higher TGF-β signal strength (>0.37 in Figure 3A), when the autoactivation of RORγt and Foxp3 eventually overrides their mutual inhibition and makes the double-expressing state the dominant phenotype of the population.

We next checked the influence of TGF-β concentration on the fractions of responding phenotypes in a population of induced cells. For various values of [TGF-β], we simulated a population of naïve CD4^+^ T cells with cell-to-cell variability. In agreement with the bifurcation analysis, RORγt^high^Foxp3^low^ and RORγt^low^Foxp3^high^ cells appeared simultaneously over an intermediate range of [TGF-β] (between ∼0.2 and ∼0.55 units). The fraction of RORγt^high^Foxp3^high^ cells increases at higher TGF-β concentrations and eventually dominates the population when [TGF-β]>0.55. In the vicinity of 0.5 units of TGF-β, the cell population is heterogeneous, with comparable fractions of all three stable phenotypes (Figure 4A lower panel).

Although this initial model accommodates the presence of dual-positive T_H_17/iT_reg_ cells, it cannot fully explain the fine regulatory effects of varying TGF-β concentrations. For example, this model predicts that double-expressing cells dominate the population when TGF-β concentration is high, and that single-expressing cells may be converted into double-expressing cells by increasing [TGF-β]. In fact, this is not necessarily true if the effects of TGF-β saturate at high [TGF-β]. To take saturation effects into account, we incorporated two intermediate signaling proteins between TGF-β and the transcription factors Foxp3 and RORγt (Figure 2B). In this case, the system can be tri-stable even at high concentrations of TGF-β, and the total conversion of single-expressing cells into double-expressing cells would not occur. Instead, co-existence of the three phenotypes in comparable fractions might be observed over a wide range of [TGF-β] (Figure 4B).

### A model with asymmetrical interactions provides a better account of the regulatory functions of TGF-β during the coupled differentiation of T_H_17 and iT_reg_ cells

We next considered an asymmetrical model in which the network topology and parameter values differ from the symmetrical model. In the model with perfect symmetry, we assumed that the inhibitions between Foxp3 and RORγt are equally strong, which is not supported by existing experimental evidence. In fact, Foxp3 is better known for its inhibitory function on IL-17, a downstream effector of RORγt, as demonstrated by Williams and Rudensky [29]. Therefore, we revised our model by removing the direct inhibition of RORγt expression by Foxp3 and adding the inhibition of IL-17 expression by Foxp3. This revised model, with broken symmetry (Figure 1C, Table 1-last column, and Figure 3C) shows some new features. First, RORγt behaves ultrasensitively in response to varying [TGF-β] because of RORγt's positive (autoregulatory) feedback loop. Secondly, Foxp3 exhibits multiple saddle-node bifurcations derived from the broken symmetries of the pitchforks. Interestingly, the four types of stable steady states observed with the symmetrical model have been retained for Foxp3, and thus for the entire system. In fact, by varying [TGF-β], it is possible to obtain all three differentiated phenotypes in significant fractions simultaneously. Doing the same analysis for the effect of [TGF-β] on the induced cell population (Figure 4C lower panel), we found that the asymmetrical model behaved similarly to the symmetrical model. At low [TGF-β], Foxp3 single-positive cells are predicted to be the dominant cell type. As [TGF-β] increases to intermediate or high levels, the RORγt single-positive cells and the double-positive cells should appear and co-exist.

These simulation results are in agreement with recently published experimental data documenting the differential effects of TGF-β on the differentiation of T_H_17 and iT_reg_ cells [16]. Indeed, at certain intermediate concentrations of TGF-β, three phenotypes in comparable fractions have been observed [16]. In addition, the maximum percentage of Foxp3 single-positive cells was observed at some lower concentration of TGF-β. As [TGF-β] was increased, the percentage of Foxp3 single-positive cells decreased, accompanied by a concordant rise in the percentage of RORγt-expressing cells [16]. At higher concentrations of TGF-β, RORγt-only cells and double-expressing cells were found to coexist in comparable percentages [15].

Our model not only validates existing published data on the coexistence of two or more phenotypes in mixed T helper cell populations but also predicts that increasing TGF-β concentration will cause the transformation of Foxp3 single-positive cells into RORγt-expressing cells. Conversely, decreasing TGF-β concentration might result in the reverse transformation.

### Our model accommodates the observed effect of IL-6 skewing T cells into a ‘RORγt-only’ phenotype

We next simulated the influence of IL-6 on this reciprocal differentiation system. In the asymmetrical model (Figure 3C), IL-6 activates STAT3, which favors production of RORγt over Foxp3. In this model, IL-6 will not trigger differentiation in the absence of TGF-β. However, IL-6 significantly increases the fraction of RORγt-only cells over a wide range of TGF-β concentrations (Figure 4A). Also, it stimulates some of the cells in the (simulated) population to produce IL-17. These results are consistent with the observations of a few groups [13], [16]. In particular, Zhou et al. observed that low level TGF-β favors the RORγt-only phenotype and IL-17 production, whereas higher concentrations of TGF-β inhibit the production of IL-17. They also reported that the decrease of IL-17 production at higher TGF-β concentration is accompanied by an increase of Foxp3-expressing cells. We see this phenomenon in our simulation, and we further suggest that the decrease of RORγt-only cells, or the increase of the double-expressing cells, accounts for the reduced production of IL-17 at high TGF-β concentration, because double-expressing cells are known to be much less effective in producing IL-17 than the RORγt-only cells, at least in this type of *in vitro* assay with TGF-β and IL-6 [15], [16]. However, Zhou et al. observed a pronounced inhibition of IL-17 production at higher TGF-β concentration even when Foxp3 expression had not been remarkably raised [16]. This discrepancy suggests that high TGF-β level may trigger Foxp3-independent repression of IL-17 production.

Both the observations by Zhou et al. and our simulations demonstrate that only a minor fraction of RORγt-only cells exhibit IL-17 production even in the presence of IL-6. In fact, this is not an idiosyncratic phenomenon. Mariani et al. recently discovered that only a subset of T_H_2 cells produce IL-4 due to cell-to-cell variability [30], suggesting that the production of lineage-specific cytokines in T helper cells can be controlled by stochastic mechanisms.

### Our model accommodates the effect of ATRA skewing T cells into a Foxp3-expressing phenotype

**Figure 5:**
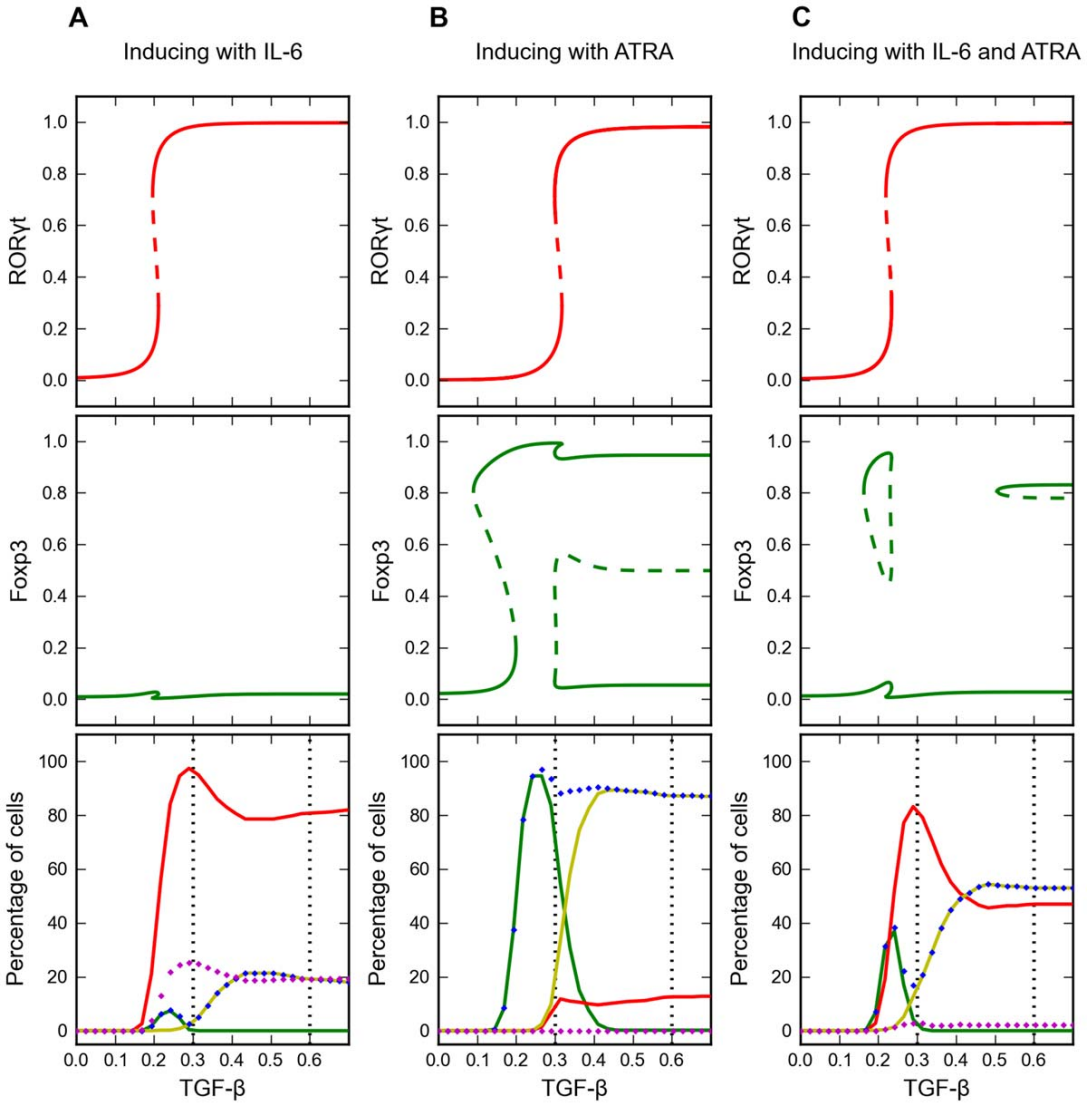
Effects of polarizing signals on the induced differentiation. Simulation of the asymmetric model (Figure 1C). Upper and middle panels: one-parameter bifurcation diagrams for the average cell. RORγt and Foxp3 steady state levels are plotted as functions of TGF-β concentration. See the legend to Figure 3 for the interpretation of the curves. **A.** Cells treated with [IL-6] = 10 units together with the indicated amount of TGF-β. **B.** Cells treated with [ATRA] = 1.5 units together with the indicated amount of TGF-β. **C.** Cells treated with [IL-6] = 10 units and [ATRA] = 1.5 units together with the indicated amount of TGF-β.

In the asymmetrical model (Figure 3C), ATRA favors production of Foxp3 over RORγt. Hence, in our simulation of TGF-β+ATRA stimulation, we found that the percentage of Foxp3-only cells and double-expressing cells significantly increased as compared to TGF-β alone (compare Figure 4B to Figure 3C). Like IL-6, ATRA did not trigger differentiation by itself. We next checked if ATRA can suppress the polarizing effect of IL-6. In our simulation, ATRA was effective in reducing the IL-6 induced production of IL-17. In addition, at high TGF-β concentration, ATRA significantly decreased the percentage of RORγt-only cells, and resulted in a population with comparable fractions of RORγt-only cells and double-expressing cells (Figure 5C). All of these simulation results are consistent with published data [13], [15], [17], [31]. Our model suggests that ATRA can significantly increase the percentage of Foxp3-only cells at intermediate TGF-β concentration, and the percentage of double-expressing cells at high TGF-β concentration.

### Our model predicts that IL-6 may reprogram iT_reg_ cells to IL-17 producing cells, while ATRA may prevent this reprogramming effect

**Figure 6:**
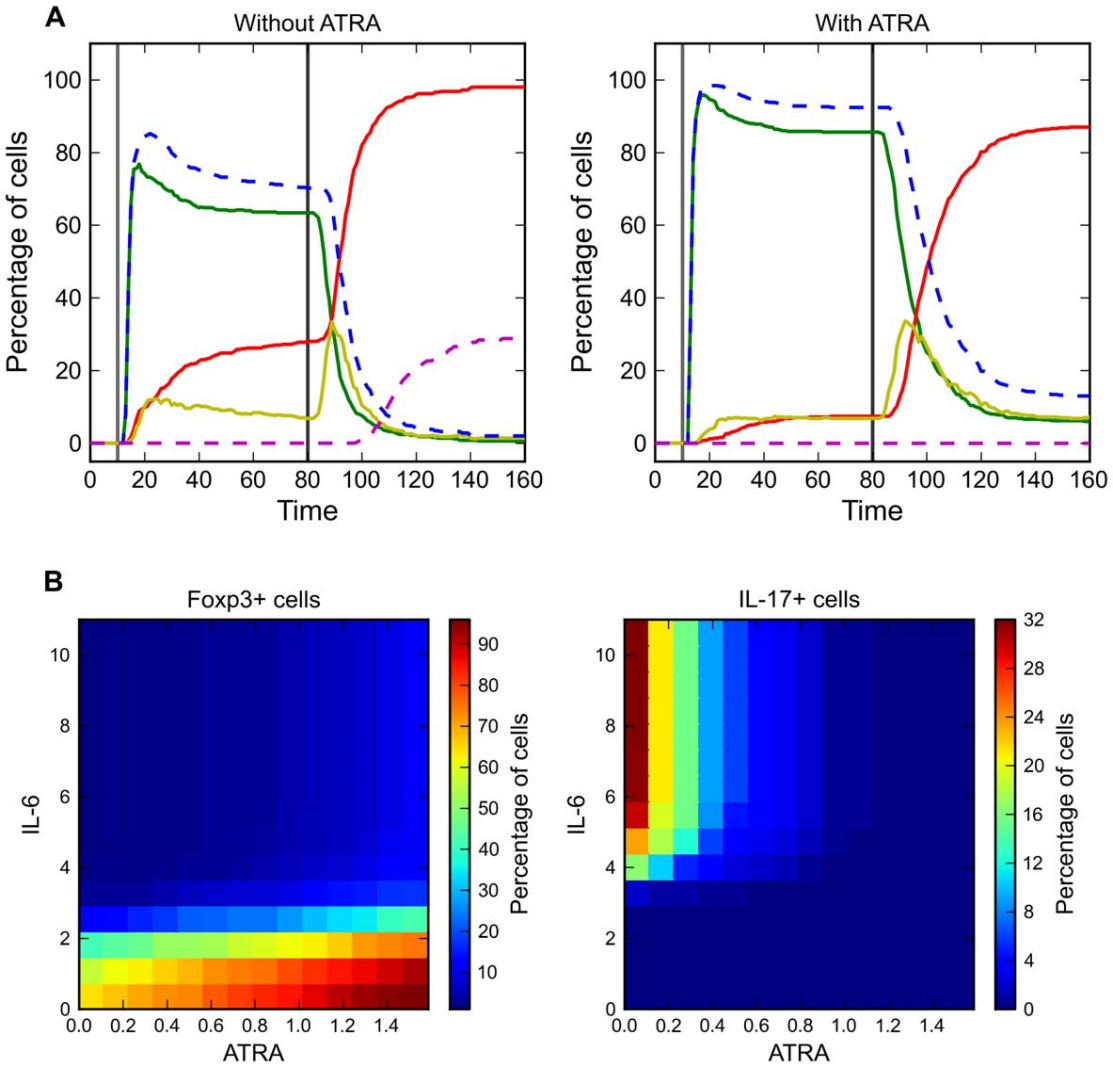
Reprogramming from iT_reg_ to T_H_17 in the presence of TGF-β. **A.** Time course trajectories of simulated reprogramming effects. 1 time unit≈1 h. [TGF-β] = 0 for t<10, and [TGF-β] = 0.28 for t>10. [IL-6] = 0 for t<80, and [IL-6] = 10 for t>80. At each time point, we plot the percentages of cells at the alternative steady states, using the same color scheme as in Figure 3. Left panel: no ATRA added. Right panel: 1.5 units of ATRA added together with TGF-β. **B.** Analysis of concentration dependencies for simulations described in Panel A. X axis: amount of IL-6 used for reprogramming. Y axis: amount of ATRA used for initial induction of differentiation. Percentages of cells at steady state are shown according to a color gradient. Left panel: percentage of Foxp3-expressing cells at steady state. Right panel: percentage of IL-17-producing cells at steady state.

With our model, we next checked whether IL-6 could reprogram differentiated iT_reg_ cells into T_H_17 cells. We first induced a population of naïve CD4^+^ T cells to differentiate into a population dominated by ‘Foxp3-only’ cells with an intermediate level of TGF-β (0.28 units). After the cells came to their Foxp3-only steady state, we raised the IL-6 signal to 10 units and continued the simulation. We found that almost all the cells expressing Foxp3 before adding IL-6 stopped producing Foxp3 upon the treatment with IL-6, and a subset of ‘RORγt-only’ cells dominated the population. A fraction of these RORγt-only cells produced IL-17 (Figure 6A, left panel).

When we induced the differentiation of iT_reg_ cells with TGF-β+ATRA and performed the same reprogramming simulation, we found that ATRA did not prevent the repression of Foxp3 expression by IL-6 significantly. However, ATRA prevented the formation of IL-17 producing cells (Figure 6A, right panel). The reprogramming capability of IL-6 and the inhibitory effect of ATRA have been observed by Yang et al. [13].

Analyzing the concentration dependence of these reprogramming effects, we found that a high level of IL-6 may exclusively down-regulate Foxp3 expression (Figure 6B, left panel) whereas a high level of ATRA may predominantly prevent IL-17 expression (Figure 6B, right panel). Interestingly, when both of these factors are present in high concentration, our model predicts that, although most cells exhibit high expression of RORγt, there are almost no IL-17-producing cells in the population. Future experimental studies are warranted to confirm these intriguing predictions.

Table 2 summarizes the observations that are in agreement with our simulation results and the testable predictions that we have made based on the bifurcation analyses and signal-response curves.

**Table 2:** Simulation results and comparisons with published experimental results.

Experimental/simulation condition	TGF-β concentration	Simulation result	Evidence
Inducing differentiation from naïve CD4^+^ T cells with TGF-β alone	Intermediate	Three phenotypes in comparable fractions	Observed [16]
	Low-intermediate	Low concentration of TGF-β gives greater percentage of Foxp3 expressing cells than intermediate concentration.	Observed [16]
	High	RORγt-only and double-expressing phenotypes in comparable fractions	Observed [15]
	Low	Foxp3-only phenotype is the major differentiated phenotype	Prediction
	From low to high	Transition from Foxp3-only phenotype to RORγt-only and double-expressing phenotypes	Prediction
	From high to low	Transition from RORγt-only or double-expressing phenotype to Foxp3-only phenotype	Prediction
Inducing differentiation from naïve CD4^+^ T cells with TGF-β and IL-6	Intermediate	Mostly RORγt phenotype, with a fraction of cells producing IL-17	Observed [16]
	High	RORγt (major fraction) and double-expressing (minor fraction) phenotypes	Observed [15]
	Low-intermediate-high	Higher concentration of TGF-β inhibits IL-17 production	Observed in more extent [16]
Inducing differentiation from naïve CD4^+^ T cells with TGF-β and ATRA	Intermediate	More Foxp3 expressing cells than with TGF-β alone	Observed [17]
	Intermediate	Foxp3-only phenotype is the major differentiated phenotype	Prediction
	High	Double-expressing phenotype is the major differentiated phenotype	Prediction
Inducing differentiation from naïve CD4^+^ T cells with TGF-β, IL-6 and ATRA	High	RORγt-only and double-expressing phenotypes in comparable fractions. IL-17 production is much lower than with TGF-β and IL-6	Observed [15]
Inducing differentiation from naïve CD4^+^ T cells to iT_reg_ cells with TGF-β, and reprogramming the differentiated iT_reg_ cells with IL-6	Intermediate	Foxp3 expressing cells are reduced, and IL-17 producing cells appear in significant fraction.	Observed [13]
Inducing differentiation from naïve CD4^+^ T cells to iT_reg_ cells with TGF-β and ATRA, and reprogramming the iT_reg_ cells with IL-6	Intermediate	Foxp3 expressing cells are reduced, and no significant number of IL-17 producing cells can be observed.	Observed [13]
	Intermediate	Most cells are in ‘poised’ state at which RORγt expression is high, but no IL-17 is produced.	Prediction

## Discussion

Previous mathematical models have shown how differentiation signals can trigger a robust switch during the development of T_H_1 or T_H_2 cells [18], [19], [20], [21], [22], [23], [24]. In particular, earlier modeling studies by Höfer et al. demonstrated how the interactions among transcription factors can create a memory for T_H_2 lineage commitment and govern the choice of T_H_1 and T_H_2 lineages [18], [19]. These studies focused on the dynamics of transcription factors within a single (average) cell, but the authors also pointed out that cell-to-cell variability in a CD4^+^ T cell population can be modeled mathematically by introducing parametric variations to the ordinary differential equations (ODEs). In addition to modeling molecular interactions, the study by Yates et al. related the dynamics of transcription factors to the phenotypic composition of T_H_1 and T_H_2 cell populations [20]. The authors built comprehensive ODE-based models which take into account cell proliferation, intercellular communication, and cell-to-cell variability. Yates et al. modeled cell-to-cell variability by variations in initial conditions, but we consider parametric variations to be a more important source of cell-to-cell variability (see Methods).

The reciprocal differentiation of T_H_17 and iT_reg_ cells, although a relatively new research field, has already been shown to exhibit many interesting and unique features, and yet it has not been studied in quantitative detail using mathematical models. The work presented here reveals some of the intriguing regulatory mechanisms of this differentiation system. We showed that the four phenotypes of cells, corresponding to four different steady states of the dynamical system, are derived from a pitchfork bifurcation with certain degree of broken symmetry. A single primary differentiation signal, TGF-β, can give rise to multiple cell types with distinct functions, while other polarizing differentiation signals, such as IL-6 as ATRA, skew the system to particular type(s) of cells. If we regard TGF-β as tossing dice for the naïve cells, those polarizing signals may load the dice, although they may not toss the dice themselves. The remarkable advantage of this system is that functionally synergic cells could be generated simultaneously in desired fractions with some simple differentiation inducers.

Our model suggests that the double-expressing phenotype is a re-stabilized co-expressing state, which should be observed in relatively late stages of cell differentiation. Previously, van den Ham and de Boer found this type of state in a similar dynamical system, although they chose parameter values to avoid this state for their system [24]. With perfectly symmetrical models, some other groups described a double-expressing state as an intermediate state before the decision making switch, corresponding to some bipotent precursor cells [27], [32], [33]. For the T_H_17-iT_reg_ paradigm, it is also possible that these double-expressing cells are at an intermediate state that should be converted into single-expressing cells at a later stage of the differentiation process. However, we do not favor this view for the following reasons. 1) A few studies have shown that the double-expressing cells are effective in repressing effector cell growth and/or secreting pro-inflammatory and anti-inflammatory cytokines [15], [34]. It is not likely that a differentiation intermediate would perform any conspicuous function in the immune system. 2) There are a few reports demonstrating the conversion from iT_reg_ cells to double-expressing cells [13], [14], or from RORγt-only cells to double-expressing cells [15], and to our knowledge it is not yet established that observable double-expressing cells can be converted into single-expressing cells. Assuming that differentiation from early stage to late stage is more readily to be observed than the ‘dedifferentiation’ process, these results indicate that the double-expressing cells might be at a differentiation stage later than the single-expressing states. 3) As shown in this report, there is a mathematical basis to support the double-expressing state appearing only at relatively high TGF-β concentration and some late differentiation stage, and the model is in accord with most published experimental observations. In addition, we are aware that the double-expressing cells are also observed for iT_reg_-T_H_1 and iT_reg_-T_H_2 paradigms [3]. Therefore, the framework presented here may be helpful for understanding iT_reg_ cells that express T-bet or GATA3 as well. Interestingly, conversion of Foxp3-expressing iT_reg_ cells to Foxp3/T-bet double-expressing cells has been reported [35]. In fact, these double-expressing cells may play very specific and indispensable roles in controlling inflammation. Chaudhry et al. have found that iT_reg_ cells require STAT3 for their suppressive function on T_H_17, and not on other lineages [36]. Koch et al. discovered that the T-bet expression is required for the function of iT_reg_ cells during T_H_1-mediated inflammation [35]. These results suggest that there are subpopulations of iT_reg_ cells expressing various master regulators of T helper cells, and they are tailored for different functions [3]. Therefore, the double-expressing cells might be terminally differentiated effectors performing specific suppressive functions. It is possible that the Foxp3-only cells, which mainly appear at low TGF-β concentration, could serve as precursors or reservoir for different terminal effectors, in addition to their general suppressive functions.

Although the detailed physiological significance of this delicate differentiation system is yet to be discovered, Lochner et al. have already demonstrated in mice that, during infections and inflammation, the number of IL-17 producing RORγt^+^ cells and double-expressing cells increased in remarkably comparable proportions [15]. This suggests the need for balance between different cell types in response to pathogenic challenges. A single differentiation network that gives rise to multiple phenotypes might be crucial for the maintenance of such balance. Furthermore, it is worth highlighting the common features shared by the T_H_17-iT_reg_ differentiation system and the differentiation control systems of hematopoietic cells and of stem cells [27], [28], [37]. Functionally, these systems have the potential to generate multiple phenotypes in a single differentiation event, and these phenotypes may play synergic roles under certain physiological conditions. In addition, it has been shown that cell-to-cell variability within clonal populations makes significant contributions to the stochasticity of lineage choice in stem cells [38]. This is also concordant with our basic assumptions.

Pitchfork bifurcations (with broken symmetry) may be the underlying mechanism generating variable phenotypes in these dynamical control systems. We will not be surprised if other cell differentiation systems possess similar properties. Recently, Heinz *et al* discovered that the ‘priming factor’ PU.1, which is required for both macrophage and B cell differentiation, is responsible for creating some of the lineage specific epigenetic markers by itself [39]. Therefore, it is possible that these priming factors not only drive the differentiation event, but also help to create a heterogeneous population of cells.

One limitation of our model is the assumption that the high concentration of TGF-β used by Lochner et al. is above the saturation concentration for TGF-β signaling [15]. We are cautious about extrapolating our model to even higher TGF-β concentration because there is no available experimental result for us to compare with. In fact, it is possible that at even higher TGF-β concentration either the RORγt-only phenotype or the double-expressing phenotype dominates the population, and the conversion between these two phenotypes might be possible by adjusting the concentration of TGF-β. Although Lochner et al. observed the conversion of RORγt-only cells into double-expressing cells at late time points of induced differentiation, we are not sure about the nature of this conversion: it could be a transition from a transient intermediate to a stable steady state; it could be a transition triggered by a slow increase of TGF-β signaling in RORγt cells, possibly mediated by paracrine signaling (see below); or it may be caused by slow fluctuations in the transcriptomes [38]. Nonetheless, when more experimental results become available, we should be able to pinpoint the missing pieces in this reciprocal differentiation system and make the mathematical model more helpful for our understanding of the system in detail.

Another limitation of this study is that we have neglected the effects of intercellular communication on the differentiation of CD4^+^ T cells. Cytokines secreted by T_H_1 and T_H_2 cells are known to influence the differentiation of neighboring T cells [40], and previous modeling work has highlighted the importance of these paracrine signaling effects [20]. Relevant to our work, the cytokines secreted by T_H_17 and iT_reg_ cells can influence the differentiation of a population of T cells, and this influence might be reflected in changes of the proportions of induced phenotypes. For example, both T_H_17 and iT_reg_ cells can produce TGF-β [41], [42], which may increase the percentage of both type of cells, or induce the transition from single-expressing cells to double-expressing cells, and this may be causative for the transition observed by Lochner et al. [15]. However, it is not yet clear how important are paracrine signals via secreted cytokines compared to exogenous cytokine signals, with respect to T_H_17 and iT_reg_ differentiation. We leave the consideration of these factors for future work.

In summary, we presented a novel mathematical model of T_H_17-iT_reg_ differentiation. Based on the model, we show how TGF-β can trigger the differentiation of naïve CD4^+^ T cells into a heterogeneous population containing RORγt-only, Foxp3-only and double-expressing cells, and how polarizing signals can skew the differentiation to particular phenotype(s). The model suggests how the conversions among different phenotypes can be guided. Additionally, the model gives a new quantitative explanation for the double-expressing cells, which should appear only at a late stage of the differentiation process. Our model provides new insights into the regulatory mechanisms that underlie the molecular control of certain immune responses.

## Methods

We constructed our mathematical model based on known interactions among key molecules in the differentiation system of T_H_17 and iT_reg_ cells. For illustrative purposes, we first consider a ‘symmetrical’ model in which the lineages of T_H_17 and iT_reg_ have identical corresponding interaction types and strengths. Then we added two intermediate proteins for transmitting TGF-β signals in this symmetrical model. Next, we modified our model so that it became asymmetrical, and we incorporated two other input signals. Using this last model, we compared our simulation results with some published experimental data and made several testable predictions.

In the symmetrical model (Figure 2A) TGF-β upregulates both RORγt and Foxp3, which has been demonstrated in a few published experiments [13], [43]. The model includes the ‘autoactivation’ of both master regulators. Although there is no evidence for direct autoactivation of RORγt and Foxp3, these relationships in our model represent known positive feedback loops in their respective pathways. One origin of these positive feedback loops is the epigenetic modifications observed in the promoter regions of RORγt and Foxp3 in their respective lineages [44], [45]. These epigenetic modifications recruit additional chromatin remodeling complexes that further stabilize those modifications and help to maintain the gene expression, thus forming positive feedback loops [46]. Additionally, master regulators can enhance their own production by autocrine effects. For example, RORγt can induce production of IL-21 and IL-23 which further stimulate the expression of RORγt, as suggested by Murphy and Stokinger [47]. The symmetric model also includes the cross-inhibition interactions between Foxp3 and RORγt. Inhibition of Foxp3 by RORγt is supported by the recent discovery that RORγt acts as a transcriptional repressor of Foxp3 by binding to its promoter [48]. Although a few reports suggest a functional inhibition of RORγt by Foxp3 [13], [16], [49], the presence of Foxp3 was shown to have no pronounced effect on the expression of RORγt [50]. Our symmetrical model includes the inhibition of RORγt by Foxp3, but we relaxed this assumption in our model with broken symmetry.

In the first version of our symmetrical model, TGF-β directly activates RORγt and Foxp3. In the second version, we added intermediate proteins between TGF-β and the master regulators. It is known that Smad2, Smad3 and Smad4 mediate the TGF-β-induced upregulation of Foxp3 [51], [52], but the Smad proteins are dispensable for upregulation of RORγt. It is still unclear how the TGF-β signal is transmitted to RORγt [52]. Thus, in Figure 1B, we introduce a generalized ‘Smad’ intermediate between TGF-β and Foxp3 and an ‘unknown intermediate’ between TGF-β and RORγt.

The model with broken symmetry also includes IL-17, which is activated by RORγt and STAT3, and deactivated by Foxp3 and ATRA [8], [13], [16], [29], [53]. As a polarizing signal, IL-6 stimulates RORγt and IL-17 production, and represses Foxp3 expression through the STAT3 pathway [54]. Conversely, ATRA upregulates Foxp3, downregulates RORγt, and inhibits IL-17 production [17], [31]. These relations are all included in our model with broken symmetry (Figure 2C).

To model the T_H_17-iT_reg_ reciprocal-differentiation system, we use a generic form of ordinary differential equations (ODEs) that describe both gene expression and protein interaction networks [55], [56], [57]. Each ODE in our model has the form:
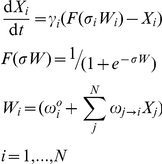


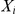
 is the activity or concentration of protein 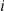. *X_i_*(*t*) changes on a time scale = 1/γ_i_. *X_i_*(*t*) relaxes toward a value determined by the sigmoidal function, *F*, which has a steepness set by 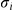. The basal value of *F*, in the absence of any influencing factors, is determined by 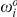. The coefficients 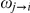 determine the influence of protein 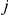 on protein 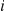. 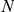 is the total number of proteins in the network. For example, the pair of ODEs for the first symmetrical model are:
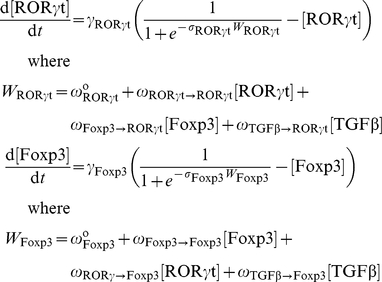
All variables and parameters are dimensionless. One time unit in our simulations corresponds to approximately 1 hour.

All simulations and bifurcation analyses were performed with PyDSTool, a software environment for dynamical systems [58]. In the Supplementary Information we provide a Python module file (Text S1) for PyDSTool that completely defines the ODEs we are solving in each case, and an example script (Text S2) to reproduce bifurcation diagrams shown in Figure 4A.

All the experimental results to which our model has been compared were obtained with differentiation assays that lasted 2–5 days, and these results are essentially consistent from one experiment to another. Thus, we assumed that the observed, differentiated cell phenotypes after 2–5 days are representative of stable steady states in our model.

We have chosen to use generic (phenomenological) ODEs instead of a more detailed kinetic model of the biochemical reaction network because we lack sufficient mechanistic and kinetic information on the molecular interactions in the T_H_17-iT_reg_ reciprocal-differentiation system. To build a detailed biochemical model, based on mass-action or Michaelis-Menten kinetics, would require us to make many assumptions on the underlying mechanism and rate constants with little or no experimental evidence to back up these assumptions. In such a case, a phenomenological model seems more appropriate to us. A similar approach has been adopted in earlier theoretical studies of T cell differentiation by Mendoza and Xenarios [22], who used a sigmoidal function similar to our F(σW), and by van den Ham and de Boer [21], who used Hill functions in place of our F(σW). To be sure that our results are not overly dependent on our mathematical approach, we have re-formulated our ‘symmetrical model without intermediates’ using Hill functions and confirmed that the model exhibits four types of stable steady states as [TGFβ] is varied. The basic features of the bifurcation diagrams and signal-response curves are similar, regardless of which formalism is used (details available upon request).

To account for cell-to-cell variability in a population, we made many simulations of the system of ODEs, each time with a slightly different choice of parameter values, to represent slight differences from cell to cell. We assumed that the value of each parameter conforms to a normal distribution with CV = 0.05 (CV = coefficient of variation = standard deviation/mean). The mean value that we specified for each parameter distribution is also referred as the ‘basal’ value of that parameter (see Table 1). In our bifurcation analysis of the dynamical system, we consider an imaginary cell that adopts the basal value for each of its parameters, and we define this cell as the ‘average’ cell. Note that none of the cells in our simulated population is likely to be this average cell, because every parameter value is likely to deviate a little (CV = 5%) from the basal value. Note, in addition, that our simulations sample a volume of parameter space around the ‘average’ cell, thereby probing the sensitivity/robustness of the differentiation process. Because we are varying all parameters simultaneously and randomly, this procedure is more indicative of robust behavior than standard sensitivity analysis, which involves estimating the partial derivative of some output property (e.g., steady state level of Foxp3) with respect to each parameter separately.

In order to simulate the induced differentiation process, we first solved the ODEs numerically with some small initial values of [RORγt] and [Foxp3] state and with [TGF-β] = 0 (and, if applicable, other input signals, e.g. IL-6 and ATRA,  = 0 as well). After a short period of time, each simulated cell will find its own, stable RORγt^low^Foxp3^low^ steady state, corresponding to a naïve CD4^+^ T cell. Next, we changed [TGF-β] (and other input signals, if applicable) to a certain positive value and continued the numerical simulation. By the end of the simulation, each cell arrives at its corresponding ‘induced’ phenotype, which might vary from cell to cell because of the parametric variability of the population. To simulate the reprogramming effect, the concentration of IL-6 was raised after the cells were stabilized in the differentiated state. We made the simple definition that a protein is expressed when its level is greater than 0.5 units.

To check the effect of TGF-β concentration on the induced phenotypes, we ran a series of simulations for a group of 1000 cells with various values of [TGF-β] and plotted the percentages of cells that adopt each terminal phenotype, in order to generate a ‘signal-response’ curve for a population of cells. Note that this signal-response curve could only represent a series of induced differentiation experiments with various TGF-β concentrations instead of a single experiment with increasing concentration of TGF-β.

Our simulations of cell-to-cell variability are based on the assumptions that each cell follows a deterministic trajectory but that cells differ from one another in the precise values of the kinetic parameters that govern the deterministic trajectory. A similar approach was adopted by Höfer et al. in their model of transcriptional regulation of T lymphocytes [18]. An alternative view of stochasticity assumes that all cells are identical in terms of kinetic constants but they follow unique stochastic trajectories because of random fluctuations in the numbers of molecules of the dynamic variables. The truth is most likely a combination of these effects (parameter variation and molecular fluctuations), but we have adopted the parameter-variation approach for several reasons. First of all, we lack the sort of molecular details (e.g., the numbers of molecules of regulatory species per cell) needed for accurate stochastic simulations of molecular fluctuations. Second, it is unlikely that T cells are identical with respect to parameter values, and there is experimental evidence to the contrary. Peripheral naïve T cells undergo a complex developmental process in the thymus, where they likely inherit many stable cell-to-cell differences, possibly because of the great diversity of T cell receptor specificities generated by VJ or V(D)J recombination. Experiments on T cell differentiation are done by selecting cells with some common characteristics, but they may nonetheless differ in many other respects. Even monoclonal populations of mammalian cells (derived from a single progenitor cell) exhibit a distribution of properties that can affect cell fate determination [38]. Nonetheless, to be sure that our results are not overly dependent on our view of cell-to-cell variability, we have re-formulated our ‘symmetrical model without intermediates’ as a pair of stochastic differential equations with additive white noise and confirmed that the SDEs generate signal-response curves similar to our results in Fig. 4A, bottom panel (details available upon request).

It is also reasonable to attribute variability among cells to different initial conditions for each simulation of the governing ODEs, as suggested by Yates et al. [20]. Since variations of initial conditions can also bias cells toward different phenotypes, we presume that this strategy will produce results similar to our own.

## Supporting Information

Text S1 **A module file that defines the ODEs for the three models.** This is a Python module file that specifies the equations and the parameter values for the three models discussed in the paper. They can be used as inputs for simulations and analyses with PyDSTool.
(TXT)

Text S2 **An example script for generating bifurcation diagrams.** This is a Python script file that produces the 1-parameter bifurcation diagram shown in Figure 4A. It requires PyDSTool and the module file that defines the ODEs (Text S1).
(TXT)
